# Reproductive Outcomes Following Ectopic Pregnancy: Register-Based Retrospective Cohort Study

**DOI:** 10.1371/journal.pmed.1001243

**Published:** 2012-06-19

**Authors:** Sohinee Bhattacharya, David J McLernon, Amanda J Lee, Siladitya Bhattacharya

**Affiliations:** Obstetric Epidemiology, Division of Applied Health Sciences, University of Aberdeen, Aberdeen, Scotland; Medical Statistics Team, Division of Applied Health Sciences, University of Aberdeen, Aberdeen, Scotland; Obstetrics and Gynaecology Group, Division of Applied Health Sciences, University of Aberdeen, Aberdeen, Scotland; London School of Hygiene and Tropical Medicine, United Kingdom

## Abstract

**Background:**

We aimed to compare reproductive outcomes following ectopic pregnancy (EP) versus livebirth, miscarriage, or termination in a first pregnancy.

**Methods And Findings:**

A retrospective cohort study design was used. Scottish national data on all women whose first pregnancy occurred between 1981 and 2000 were linked to records of a subsequent pregnancy. The exposed cohort comprised women with an EP in their first pregnancy. There were three unexposed cohorts: women with livebirth, miscarriage, and termination of their first pregnancies. Any differences in rates of second pregnancy, livebirth, EP, miscarriage, or terminations and complications of a second ongoing pregnancy and delivery were assessed among the different exposure groups. A total of 2,969 women had an initial EP; 667,299 had a livebirth, 39,705 women miscarried, and 78,697 terminated their first pregnancies. Women with an initial EP had an increased chance of another pregnancy within 2 years (adjusted hazard ratio (AHR) 2.76 [95% CI 2.58–2.95]) or after 6 years (AHR 1.57 [95% CI 1.29–1.91]) compared to women with a livebirth. In comparison with women with an initial miscarriage, women who had an EP had a lower chance of a second pregnancy (AHR 0.53 [95% CI 0.50–0.56]). Compared to women with an initial termination, women with an EP had an increased chance of a second pregnancy (AHR 2.38 [95% CI 2.23–2.55]) within 2 years. Women with an initial EP suffered an increased risk of another EP compared to women with a livebirth (AHR 13.0 [95% CI 11.63–16.86]), miscarriage (AHR 6.07 [95% CI 4.83–7.62]), or termination (AHR 12.84 [95% CI 10.07–16.37]). Perinatal complications in a pregnancy following EP were not significantly higher than those in primigravidae or in women with a previous miscarriage or termination.

**Conclusion:**

Women with an initial EP have a lower chance of conception than those who miscarry but an increased risk of a repeat EP in comparison with all three comparison groups. A major limitation of this study was the inability to separate women using contraception from those who were intending to conceive.

*Please see later in the article for the Editors' Summary*

## Abstract 2

Using Scottish national registry data, Sohinee Bhattacharya and colleagues investigate pregnancy outcomes following ectopic pregnancy in comparison to livebirth, miscarriage, or termination in a first pregnancy.

## Editors' Summary

**Background:**

An ectopic pregnancy occurs when the embryo (fertilized egg) implants outside the uterine cavity, usually in the fallopian tubes but sometimes in the cervix, ovaries, or abdomen. The prevalence for this condition is between 1%–2% of all pregnancies, and risk factors are thought to include pelvic infection, smoking, previous pelvic surgery, and use of certain types of intrauterine contraceptive devices. Ectopic pregnancies are potentially life threatening because as the fetus grows, it can lead to tubal rupture and abdominal bleeding—for example, in the UK, ectopic pregnancies are responsible for almost three-quarters of early pregnancy-related deaths. However, due to improvements in early diagnosis, in high income countries, deaths from ectopic pregnancies have become increasingly rare.

**Why Was This Study Done?:**

Having an ectopic pregnancy can have serious implications for future fertility and subsequent pregnancies but to date, there is little information on reproductive outcomes in women who have had an ectopic pregnancy. So in this study, the researchers used a population-based cohort of women in Scotland to examine future reproductive outcomes in women who had an initial ectopic pregnancy and then compare these outcomes to those in women following a successful (live birth) or unsuccessful (miscarriage or termination) intrauterine pregnancy.

**What Did The Researchers Do And Find?:**

The researchers used a national database (The Scottish Morbidity Record) and hospital discharge information to identify women who had ectopic pregnancies, miscarriages, terminations, or on-going pregnancies between 1981–2000. Then, using unique linking identifiers, they were able to examine the outcomes of subsequent pregnancies and conducted a statistical analysis to investigate whether the first pregnancy outcome had any effect on second pregnancy outcomes.

The researchers found that during the time period studied, in their first pregnancy, 2,969 women had an ectopic pregnancy, 39,705 women miscarried, 78,697 women underwent termination, and the majority, 667,299, gave birth to a live infant. The researchers then found that compared to women with an initial live birth, women with an ectopic pregnancy were 2.76 times more likely to conceive a second pregnancy within two years. However, compared to women whose first pregnancies ended in miscarriage, women with an initial ectopic pregnancy were significantly less likely to conceive a second time but had an increased chance of a second pregnancy within two years compared to women who terminated their first pregnancy. Importantly, the researchers found that women with an initial ectopic pregnancy had a higher risk of a further ectopic pregnancy compared to all the other groups of women. Furthermore, these women had a significantly higher risk of preeclampsia, preterm delivery, and emergency cesarean delivery in their next continuing pregnancy compared to women who had a previous live birth. However, these risks were not significantly higher than women who had an early loss in a first pregnancy.

**What Do These Findings Mean?:**

These findings suggest that women with an initial ectopic pregnancy have a lower chance of conception than those who miscarry and also have an increased risk of a repeat ectopic pregnancy compared to women who experience miscarriage, termination, or a live birth in their first pregnancy. However, as the researchers did not have any information on contraception use, a major limitation of this study is the inability to separate women using contraception from those who were intending to conceive—women who experienced an ectopic pregnancy may not want to conceive again after a traumatic experience rather than being unable to conceive because of tubal damage. However, the results of this study may help doctors to counsel women with an ectopic pregnancy at the time of initial diagnosis and treatment, and in those willing to conceive again, offer follow-up to discuss future fertility and possible risks of subsequent pregnancy. Further research will help to investigate whether the site of ectopic pregnancy affects future reproductive outcomes.

**Additional Information:**

Please access these Web sites via the online version of this summary at http://dx.doi.org/10.1371/journal.pmed.1001243.

- The American Pregnancy Association and the UK National Health Service (NHS) Choices give information on ectopic pregnancy
- The UK nonprofit organization Ectopic Pregnancy Trust provides support for individuals affected by ectopic pregnancy

## Introduction

An ectopic pregnancy (EP) occurs when a fertilised ovum implants at a site outside the uterine cavity. The commonest location is within the fallopian tube, and the condition remains a significant cause of morbidity and mortality due to the associated risks of tubal rupture and intra-abdominal haemorrhage. Between 2003 and 2005, EPs accounted for 72% of early pregnancy deaths in the United Kingdom [1]. Suggested risk factors include pelvic infection, smoking, previous pelvic surgery, sterilisation, use of certain types of intrauterine contraceptive devices (IUCD), a previous ectopic pregnancy, and older maternal age [2],[3]. In recent years, early diagnosis has meant that maternal deaths due to EP have become increasingly rare in the developed world and the clinical emphasis has now shifted to preservation of fertility [4]. To assess the existing evidence regarding reproductive outcomes following ectopic pregnancy, we conducted a literature search in Ovid MEDLINE (1966–2008), EMBASE (1996–2008), and CINAHL (1986–2008) using the search terms “ectopic=", “pregnancy or gestation=", and “outcomes=". Several papers on risk factors for tubal EP were identified [5],[6], but few studies explored pregnancy outcomes following an ectopic gestation comparing them to those following intrauterine pregnancies. Two studies specifically addressing this issue reported conflicting results. Hassan and Killick [7] reported a 3-fold increase in conception rates following EP, while a much larger study based on the EP registry in Auvergne, France [8] found no independent effect of ruptured tubal EP on subsequent fertility. In France, the Auvergne registry collects data on all ectopic pregnancies occurring in a geographically defined area and several articles based on this register have been published. These articles, although providing an insight into the epidemiology of EP, do not present comparisons with intrauterine pregnancies.

The relationship between fertility and EP is of interest to researchers and clinicians because the same underlying pathology, for example tubal damage, may result in both infertility and ectopic pregnancy. The factor underlying reduced fertility and recurrence of EP is possibly pelvic inflammatory disease (PID). Westrom et al. [9] found that rates of EP in women diagnosed with PID by laparoscopy were 9.1% versus 1.4% in those without PID. Moreover, the shift in management strategies for EP towards tubal conservation may have improved fertility but contributed to increased recurrence.

Our aim was to investigate, in a population-based cohort of women, reproductive outcomes after an initial EP and compare them to outcomes following successful or unsuccessful intrauterine pregnancies.

## Methods

### Ethics Statement

Formal ethical approval was not considered necessary by the North of Scotland Research Ethics Service. Approval was obtained from the Privacy Advisory Committee of the Information and Services Division of NHS Scotland.

### Databases and Variables

The Scottish Morbidity Record obtains information on clinical and demographic characteristics and outcomes for all women discharged from Scottish hospitals. Information maintained by the Information and Services Division (ISD) of NHS Scotland offers unique opportunities to understand and explore the epidemiology of EP in Scotland over a defined period of time. A register-based cohort study design was employed here, using routinely collected data from the database of Scottish Morbidity Records (SMR). Data were extracted on all women who had an EP, a miscarriage, a termination, or an ongoing pregnancy and delivery in their first pregnancy between 1981 and 2000 recorded in these databases (SMR01 and SMR02). The two pregnancy events were linked by internal data linkage procedures using unique identifiers. Subsequently, identifiers were removed and an anonymised dataset was provided by the ISD to the researchers.

The Scottish Morbidity Record obtains information on clinical and demographic characteristics and outcomes for all women discharged from Scottish hospitals. Information maintained by the Information and Services Division (ISD) of NHS Scotland offers unique opportunities to understand and explore the epidemiology of EP in Scotland over a defined period of time. A register-based cohort study design was employed here, using routinely collected data from the database of Scottish Morbidity Records (SMR). Data were extracted on all women who had an EP, a miscarriage, a termination, or an ongoing pregnancy and delivery in their first pregnancy between 1981 and 2000 recorded in these databases (SMR01 and SMR02). The two pregnancy events were linked by internal data linkage procedures using unique identifiers. Subsequently, identifiers were removed and an anonymised dataset was provided by the ISD to the researchers.

The following variables were obtained by matching SMR01 and SMR02 datasets:

demographic details: age at first and second pregnancy, smoking status (data available for a subset of women), social class (assessed using Carstairs' index of deprivation) [10]; details of EP: previous history of EP, date of event; reproductive outcomes: intrauterine pregnancy, repeat ectopic, miscarriage, termination, stillbirth.

The data were coded according to the International Classification of Diseases 9th and 10th Revisions and stored in the SMR databases.

### Second Pregnancy Outcomes

In a retrospective cohort study, reproductive outcomes of second pregnancy in women who had an EP in their first pregnancy (exposed group), were compared with those in three unexposed groups: (1) women who had a live birth in their first pregnancy (unexposed group A); (2) women who had a miscarriage in their first pregnancy (unexposed group B); (3) women who had an abortion (termination of pregnancy) in their first pregnancy (unexposed group C).

### Perinatal Complications in Second Ongoing Pregnancy

A subgroup analysis was carried out in women who had a second continuing intrauterine pregnancy leading to delivery in order to assess the risks of maternal and perinatal complications following an ectopic first pregnancy. Again, a cohort study design was used, where the exposed cohort comprised women who had an EP in their first pregnancy and then had an ongoing pregnancy leading to delivery. The unexposed cohorts consisted of women who had an ongoing second pregnancy leading to delivery but had previously had a miscarriage, a termination, or a live birth in a first pregnancy. A fourth comparison group consisted of primigravid women with an ongoing pregnancy leading to delivery as the risks of complications like preeclampsia were dependent on parity.

The proportion of women with preeclampsia, placenta praevia, and placental abruption in the exposed cohort were compared with those in each of the four unexposed cohorts. Also, perinatal outcomes such as preterm delivery and low birth weight were compared across the groups, as was mode of delivery.

### Statistical Analysis

Demographic variables including age at pregnancy event, social class, and smoking status were compared in exposed and unexposed cohorts using appropriate univariate tests (analysis of variance for continuous variables and Pearson's chi- square test for categorical variables).

Survival analysis was conducted to investigate whether first pregnancy outcome had an effect on time to the second pregnancy outcomes—any second pregnancy, miscarriage, EP, termination, or stillbirth. Kaplan-Meier survival curves of time to second pregnancy from the date of first pregnancy outcome (i.e., ectopic, live birth, miscarriage, termination) were produced. Cox's proportional hazards models were used to calculate the hazard ratio (HR) with 95% CIs of each of these outcomes following different first pregnancy outcomes. The survival models were adjusted for age at first pregnancy, social class, and year of admission for first pregnancy. The proportional hazards assumption was checked by plotting curves of the log of the negative log of the survival function against log time for each first pregnancy outcome. If all the curves were approximately parallel then the proportional hazards assumption is satisfied. Where the proportionality assumption failed, separate models were fitted stratified by time using time points informed by where the Kaplan-Meier curves cross.

As the exact timing of the episode of pregnancy complication was not available, survival analysis was not appropriate. Hence, binary logistic regression was used to produce crude odds ratios (ORs) with 95% CIs for each of the obstetric complications in an ongoing pregnancy. Models were subsequently adjusted for potential confounders identified on univariate analysis that were significant at the 5% level. As the data spanned 25 y, the year of delivery was included as a covariate in the models to account for changes in coding criteria and medical practice over time.

### Missing Data

Some potential confounding variables like body mass index (BMI), site of ectopic pregnancy, and medical management of ectopic pregnancy were not universally recorded in the dataset and therefore could not be included in the analysis. Data were also unavailable regarding the use of contraception and pregnancy intent. We therefore had to make broad assumptions that women with a live baby would be more likely to use contraception in the short term for birth spacing than those without a live birth in order to interpret our findings. Other variables were available in a subset of women (for example, smoking status was missing in 62%). As a sensitivity analysis, we ran the Cox's and logistic regression models both with (incorporating only those with complete smoking data) and without the smoking variable. Small numbers of women with missing data for other variables such as Carstairs' deprivation category (3.5%), birthweight (3.7%), or gestational age (4.2%) were not included in the analysis.

The report is presented in accordance with STROBE guidelines (Text S1).

## Results

After excluding women with improbable gestational age and interpregnancy intervals of less than or equal to 4 wk (*n* = 165), the dataset included 2,969 women who had an ectopic first pregnancy, 667,299 women with an initial live birth, 39,705 with a miscarriage, and 78,697 women who underwent termination of their first pregnancy.

### Baseline Characteristics

The socio-demographic characteristics of women with a previous ectopic pregnancy were compared with those with a previous live birth, miscarriage, and termination in Table 1. Women who had experienced an initial ectopic pregnancy tended to be older, and less socially deprived than women who had a miscarriage, termination, or live birth in their first pregnancies.

**Table 1:** Comparison of baseline characteristics of women with ectopic and intrauterine first pregnancies.

Characteristics	Outcome of First Pregnancy				
	Ectopic (*n* = 2,969)	Livebirth (*n* = 667,299)	Miscarriage (*n* = 39,705)	Termination (*n* = 78,697)	*p*-Value
Mean age in years (SD)	27.75 (5.70)	25.79 (5.47)	26.27 (6.37)	22.63 (6.46)	<0.001
Median follow up time in years (IQR)	5.21(3.17)	6.43 (3.41)	3.78(2.17)	5.79 (2.94)	
Carstairs deprivation category					<0.001
Least deprived	980 (33.0)	176,310 (26.4)	11,240 (28.3)	18,683 (23.7)	
Moderate	1,468 (49.4)	331,856 (49.7)	22,286 (56.1)	29,172 (37.1)	
Most deprived	420 (14.1)	127,201 (19.3)	5,179 (13.0)	29,239 (37.2)	
Smoking status					<0.001
Non-smoker	440 (14.8)	149,257(22.4)	6,435 (16.2)	10,180 (12.9)	
Smoker/ex-smoker	372 (12.5)	122,566 (18.4)	5,890 (14.8)	11,055 (14.1)	
Missing	2,157 (72.6)	395,476 (59.3)	27,380 (69.0)	57,462 (73.0)	

IQR, interquartile range; SD, standard deviation.

doi:10.1371/journal.pmed.1001243.t001

### Second Pregnancy Outcomes

Outcomes of a second pregnancy following an initial EP, live birth, miscarriage, and termination are listed in Table 2. Women with a live birth were least likely to have a second pregnancy (55%), while those with a miscarriage were most likely to have one (74.9%). However, when they did conceive a second time, women who had an initial live birth were most likely to have a second live birth (304,143/367,303 [82.8%]). When they conceived a second time, women with an initial EP had the highest chance of another EP compared to all other groups (144/1,870 [7.7%]).

**Table 2:** Comparison of reproductive outcomes of second pregnancy between women with ectopic and intrauterine first pregnancies.

Outcomes of Second Pregnancy	Outcome of First Pregnancy^a^			
	Ectopic*n* = 2,969	Livebirth*n* = 667,299	Miscarriage*n* = 39,705	Termination*n* = 78,697
No second pregnancy	1,099 (37.0)	299,996 (45.0)	9,978 (25.1)	26,245 (33.3)
Any second pregnancy	1,870 (63.0)	367,303 (55.0)	29,727 (74.9)	52,452 (66.7)
Live birth from second pregnancy^b^	1,455 (49.0)	304,143 (45.6)	24,201 (61.0)	39,007 (49.6)
Ectopic second pregnancy^b^	144 (4.9)	2,391 (0.4)	244 (0.6)	365 (0.5)
Miscarriage in second pregnancy^b^	154 (5.2)	23,018 (3.4)	3,577 (9.0)	3,407 (4.3)
Termination of second pregnancy^b^	103 (3.5)	36,446 (5.5)	1,511 (3.8)	9,411 (12.0)
Stillbirth in second pregnancy^b^	14 (0.5)	1,305 (0.2)	194 (0.5)	262 (0.3)

a Values expressed as *n* (%).

b Calculated as proportion of women who had a second pregnancy.

doi:10.1371/journal.pmed.1001243.t002

**Figure 1:**
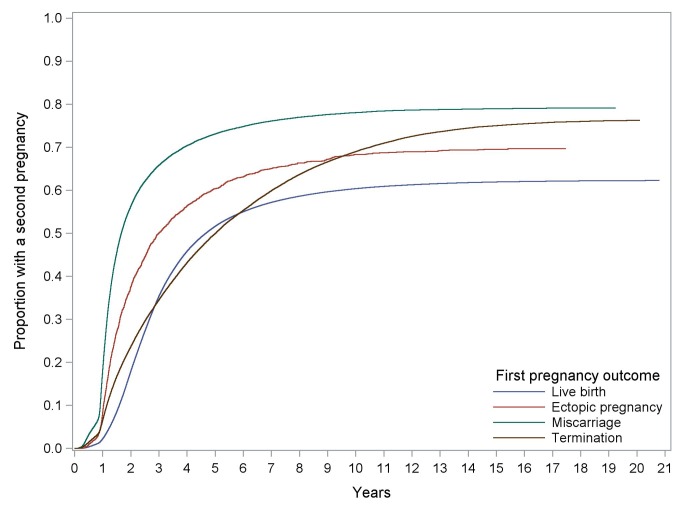
Kaplan-Meier curves of time to any second pregnancy following different first pregnancy outcomes.

Kaplan-Meier curves of time to a second pregnancy following alternative outcomes in the first pregnancy are presented in Figure 1. There was evidence of non-proportionality from the survival curves and therefore the Cox models were fitted for the following time periods: ≤2 y, >2–6 y, and >6 y. For the >2–6-y time period, women who had any second pregnancy during the first 2 y (i.e., the first time period) were excluded from the analysis. The same exclusion was applied to the >6-y time period. Women who had an ectopic first pregnancy were approximately two-and-a-half times more likely to have a second pregnancy within 2 y than those who had an initial live birth (adjusted hazards ratio [AHR] 2.76, 95% CI 2.58–2.95). Taking 2 y following their first pregnancy as the starting point, women who had an ectopic first pregnancy were no more likely to have a second pregnancy during the following 4 y (i.e., up to 6 y since first pregnancy) than women who had an initial live birth. However, from 6 y after their first pregnancy, they were approximately 50% more likely to have a second pregnancy than women who had a live birth.

Compared with women who had a miscarriage in their first pregnancy, women who had an ectopic first pregnancy were only half as likely to have a second pregnancy during the next 2 y. However, the chance of a second pregnancy in 2 to 4 y was no different in the two groups. From 6 y post first pregnancy, women with an ectopic first pregnancy were almost 50% more likely to have a second pregnancy than women who miscarried their first.

Similarly, women who had an ectopic first pregnancy were approximately two-and-a-half times more likely to have a second pregnancy within 2 y than those who terminated their first pregnancy (AHR 2.38, 95% CI 2.23–2.55) (see Table 3). Taking 2 y post first pregnancy as the origin, women who had an ectopic first pregnancy were 62% more likely to have a second pregnancy within the next 4 y than women who had an initial termination.

**Table 3:** Hazard ratios (95% CI) of any second pregnancy following ectopic versus other first pregnancy outcomes.

Comparison of 1st Pregnancy Outcome	Time to Second Pregnancy (y)^a^					
	≤2		>2–6		>6	
	Unadjusted	Adjusted^b^	Unadjusted	Adjusted^b^	Unadjusted	Adjusted^b^
Ectopic versus livebirth	2.51 (2.36–2.66)^c^	2.76 (2.58–2.95)^c^	0.88 (0.82–0.95)^c^	1.01 (0.93–1.10)	1.13 (0.96–1.34)	1.57 (1.29–1.91)^c^
Ectopic versus miscarriage	0.53 (0.50–0.56)^c^	0.57 (0.53–0.61)^c^	0.95 (0.88–1.03)	1.06 (0.97–1.16)	1.05 (0.88–1.25)	1.44 (1.17–1.78)^c^
Ectopic versus termination	1.77 (1.66–1.88)^c^	2.38 (2.23–2.55)^c^	1.05 (0.97–1.13)	1.62 (1.49–1.77)^c^	0.35 (0.29–0.41)^c^	0.95 (0.78–1.16)

a Due to evidence of violation of the proportional hazards assumption, Cox models were fitted for different time periods of follow-up. Women who had an event in the first 2 y after their first pregnancy were excluded from the >2–6-y time period analysis. Likewise, women who had an event ≤6 y after their first pregnancy were excluded from the >6-y time period analysis.

All hazard ratios have been adjusted for age at first pregnancy, social class, and year of first pregnancy.

c Statistically significant hazard ratios.

doi:10.1371/journal.pmed.1001243.t003

### Ectopic, Miscarriage, Termination, And Stillbirth In The Second Pregnancy

Table 4 presents the hazards ratios and 95% CIs of pregnancy loss following EP versus different types of intrauterine first pregnancies. Compared to women who had a first live birth, the risk of a second ectopic was 13 times higher after an initial EP (AHR 13.0, 95% CI 11.63–16.86). Risks of miscarriage (AHR 1.57, 95% CI 1.32–1.87) and stillbirth (AHR 2.75, 95% CI 1.52–4.97) were higher after an initial ectopic, but the chance of having a termination in the next pregnancy was reduced (AHR 0.66, 95% CI 0.53–0.83).

**Table 4:** Hazard ratios (95% CI) of second pregnancy outcomes following ectopic versus other first pregnancy outcome.

Second Pregnancy Outcome	1st Ectopic Versus 1st Livebirth		1st Ectopic Versus 1st Miscarriage		1st Ectopic Versus 1st Termination	
	Unadjusted	Adjusted^a^	Unadjusted	Adjusted^a^	Unadjusted	Adjusted^a^
Ectopic pregnancy	15.75 (13.31–18.63)^b^	13.0 (11.63–16.86)^b^	6.94 (5.65–8.55)^b^	6.07 (4.83–7.62)^b^	20.93 (17.17–25.51)^b^	12.84 (10.07–16.37)^b^
Miscarriage	1.76 (1.50–2.06)^b^	1.57 (1.32–1.87)^b^	0.55 (0.46–0.64)^b^	0.51 (0.43–0.61)^b^	2.13 (1.81–2.51)^b^	1.41 (1.18–1.70)^b^
Termination	0.72 (0.59–0.88)^b^	0.66 (0.53–0.83)^b^	0.79 (0.65–1.97)	0.73 (0.58–1.92)	0.46 (0.38–0.56)^b^	0.35 (0.28–0.44)^b^
Stillbirth	2.96 (1.75–5.00)^b^	2.75 (1.52–4.97)^b^	0.82 (0.47–1.40)	0.71 (0.38–1.31)	2.79 (1.63–4.80)^b^	2.28 (1.21–4.29)^b^

a All hazard ratios have been adjusted for age at first pregnancy, social class, and year of first pregnancy.

b Statistically significant hazard ratios.

doi:10.1371/journal.pmed.1001243.t004

In comparison with an initial miscarriage, the risk of a further EP was more than six times higher (AHR 6.07, 95% CI 4.83–7.62), but that of a miscarriage was less following an initial EP (AHR 0.51, 95% CI 0.43–0.61).

Compared to women who terminated their first pregnancy, women with an initial EP faced more than 12 times higher risk of a further ectopic, 41% increased risk of a miscarriage, more than double the risk of a stillbirth, but a reduced risk of terminating a second pregnancy (AHR 0.35, 95% CI 0.28–0.44).

### Perinatal Complications In A Second Pregnancy

Table 5 shows the proportion of maternal and perinatal complications in women with ongoing pregnancies following an initial EP, live birth, or termination. The ORs with 95% CIs of these complications are presented in Table 6. Compared to women with a previous live birth, an initial ectopic pregnancy predisposed women to higher odds of preeclampsia (adjusted OR [AOR] 2.19, 95% CI 1.90–4.40), preterm delivery (AOR 1.84, 95% CI 1.34–2.52), and delivery by emergency caesarean section (AOR 3.93, 95% CI 3.11–4.97).

**Table 5:** Comparison of maternal and perinatal complications in the next ongoing pregnancy following different first pregnancies.

Complications of Second Pregnancy	Ectopic *n* = 1,455	Livebirth *n* = 304,143	Miscarriage *n* = 24,201	Termination *n* = 39,007	Primigravidae *n* = 299,417
Preeclampsia	74 (4.7)	6,621 (2.2)	1,242 (4.6)	1,508 (3.7)	12,662 (4.2)
Placenta praevia	13 (0.8)	1,991 (0.7)	246 (0.9)	247 (0.6)	2,104 (0.7)
Abruptio placentae	63 (4.0)	9,151 (3.0)	1,135 (4.2)	1,704 (4.2)	10,538 (3.5)
Preterm delivery	128 (8.1)	13,871 (4.6)	2,194 (8.2)	3,042 (7.6)	19,176 (6.4)
Low birth weight	114 (7.2)	13,409 (4.4)	2,226 (8.3)	3,024 (7.5)	20,950 (7.0)
Elective caesarean section	80 (5.0)	21,611 (7.1)	1,472 (5.5)	1,523 (3.8)	16,609 (5.5)
Emergency caesarean section	241 (15.2)	17,694 (5.8)	3,937 (14.6)	5,301 (13.2)	36,798 (12.3)

Values expressed as *n* (%).

doi:10.1371/journal.pmed.1001243.t005

**Table 6:** Unadjusted and adjusted odds ratios (95% CIs) for maternal and perinatal complications in second pregnancy.

Complications of Second Pregnancy	Ectopic Versus Livebirth	Ectopic Versus Primigravidae	Ectopic Versus Termination	Ectopic Versus Miscarriage
Preeclampsia				
Unadjusted	2.20 (1.74–2.78)^a^	1.11 (0.88–1.40)	1.01 (0.99–1.02)	0.99 (0.78–1.26)
Adjusted^b^	2.19 (1.90–4.40)^a^	1.21 (0.88–1.61)	1.06 (0.92–1.22)	0.98 (0.70–1.36)
Placenta praevia				
Unadjusted	1.26 (0.73–2.17)	1.17 (0.67–2.02)	1.01 (0.99–1.04)	1.12 (0.64–1.96)
Adjusted^b^	1.31 (0.49–3.49)	0.81 (0.38–1.71)	1.17 (0.43–3.20)	1.11 (0.51–2.39)
Abruption				
Unadjusted	1.34 (1.04–1.72)^a^	1.13 (0.88–1.46)	0.99 (0.98–1.01)	1.07 (0.82–1.38)
Adjusted^b^	1.36 (0.85–2.19)	0.84 (0.59–1.18)	0.98 (0.62–1.56)	1.12 (0.77–1.62)
Preterm delivery				
Unadjusted	1.84 (1.53–2.20)^a^	1.28 (1.07–1.53)^a^	1.07 (0.84–1.37)	1.01 (0.84–1.22)
Adjusted^b^	1.84 (1.34–2.52)^a^	1.14 (0.83–1.58)	1.02 (0.74–1.40)	0.99 (0.78–1.24)
Low birth weight				
Unadjusted	1.68 (1.39–2.04)^a^	1.56 (1.09–2.24)^a^	0.95 (0.74–1.23)	1.17 (0.96–1.42)
Adjusted^b^^,^ c	1.20 (0.77–1.86)	1.03 (0.85–1.24)	0.86 (0.61–1.22)	1.07 (0.92–1.46)
Elective caesarean section				
Unadjusted	1.01 (0.75–1.36)	0.93 (0.69–1.26)	1.47 (1.08–2.00)^a^	1.04 (0.90–1.20)
Adjusted^b^	1.04 (0.71–1.52)	0.91 (0.62–1.32)	1.23 (0.83–1.82)	0.99 (0.89–1.24)
Emergency caesarean section				
Unadjusted	3.72 (3.08–4.48)^a^	1.27 (1.06–1.52)^a^	1.27 (1.05–1.54)^a^	1.13 (0.87–1.46)
Adjusted^b^	3.93 (3.11–4.97)^a^	1.08 (0.86–1.35)	1.12 (0.88–1.42)	1.04 (0.81–1.48)

a Statistically significant odds ratios.

b All odds ratios have been adjusted for maternal age, smoking, Carstairs category, and year of delivery.

c Low birth weight also adjusted for gestational age.

doi:10.1371/journal.pmed.1001243.t006

The AORs of maternal and perinatal complications were not significantly higher following an EP in comparison with primigravid women or those with a previous miscarriage or termination.

## Discussion

### Summary Of Findings

Compared to women with an initial live birth, women with an EP were 2.76 times more likely to conceive a second pregnancy within 2 y and just as likely after 2 to 6 y. Women with an initial ectopic pregnancy were significantly less likely to conceive a second time compared to women whose first pregnancies ended in a miscarriage. Compared to women with an initial termination, women with an EP had an increased chance of a second pregnancy within 2 y. They also faced a higher risk of a further ectopic pregnancy compared to all defined comparator groups.

In comparison with women who had a previous live birth, those with an initial EP faced a significantly higher risk of preeclampsia, preterm delivery, and emergency caesarean delivery in their next continuing pregnancy. However, these risks were not significantly higher than those faced by primigravid women or those who had an early pregnancy loss in a first pregnancy.

### Strengths And Limitations

To our knowledge, this study is the first population-based comparison of reproductive outcomes following ectopic and intrauterine pregnancy. Previous registry-based studies have tended to concentrate on risk factors [5],[6],[11] rather than on fertility outcomes following EP. Reports on reproductive performance after ectopic gestation have been limited by small sample sizes and, in the absence of a suitable unexposed cohort, have tended to make internal comparisons within groups of women with EP [12],[13]. In reality, there is no ideal comparison group for women with first ectopic pregnancies. Our analysis compared the reproductive outcomes of women who had an initial EP with women who had a previous successful pregnancy, women with previous spontaneous and induced pregnancy loss, and those without any previous pregnancies. While parous women have previous experience of pregnancy and labour, primigravidae have experienced neither. Those who have had an early loss of first pregnancy (ectopic, miscarriage, or termination) behave like “virtual primigravidae=" in terms of their outcomes in the next continuing pregnancy and are comparable amongst themselves or with primigravid women. We have presented the risks of reproductive complications following ectopic pregnancy relative to all groups—thus adding validity to our findings. The presence of a large number of first ectopic pregnancies recorded in the Scottish database, allowed us to focus solely on reproductive outcomes following an initial ectopic pregnancy, which we felt was the relevant clinical question.

For the survival analysis of time to second pregnancy, the proportional hazards assumption for the Cox regression model was not met. This is evident from Figure 1 since the termination curve is not approximately parallel with the others. As a result it was decided to fit separate Cox models for different time periods. The time periods were chosen in a subjective manner so that within each model the proportionality was better than for the model using the whole follow-up period. The plots of the log of the negative log of the survivor function against log time for each of these models demonstrated that the proportionality assumption was adequately met for each of them. As with most statistical assumptions, it is rarely the case that the proportionality assumption is fully met. Using the model for the whole time period would have given us an average effect for the covariates over the whole time period, which is still useful. However, separate models for different stratified time periods gives us more information about short-term follow-up (i.e., within 2 y) and about longer term follow-up.

Cox regression was conducted to examine the effect of different outcomes from the first pregnancy on time to a miscarriage, EP, termination, or stillbirth in the second pregnancy. In this analysis, only women with a second pregnancy were included in the models since they were the “at risk=" population. Furthermore, for each outcome, women with any of the other outcomes were censored. In doing this we have assumed that censoring due to these other outcomes is independent of the occurrence of the outcome of interest. The validity of this assumption will be explored in a future paper that will account for these “competing outcomes=" [14].

Most of the limitations of this study arise from lack of complete data on variables such as smoking status [15],[16], as well as anatomical site [16], outcome [17], type of management [18]–[21] of EP, which are potentially associated with future reproductive outcomes. The prevalence of smoking during pregnancy has decreased from 25.4% in 2001 to 18.8% in 2010 in Scotland (www.isdscotland.org). These data, however, are only applicable to women who had deliveries—at or near term and exclude women with early pregnancy loss. Data on maternal body weight were universally unavailable in our dataset and it was impossible to predict what effect this would have had, had we been able to adjust for it. Literature is sparse regarding the effect of obesity on the prevalence of EP, but studies have shown an increased risk of recurrence in obese women who miscarry [22]. The prevalence of other potential confounders like assisted reproductive technology and medical management of EP in the study population are more difficult to obtain. Reports published show that on average, 1% of the total number of pregnancies in the UK is conceived through assisted reproduction (www.hfea.gov.uk). Despite unavailability of data, we were able to use some of the variables in the analysis of a subset of data where the information was complete; but this severely limited the power of any findings. Moreover, the data spanned 25 y, which have witnessed significant changes in clinical management that are likely to influence the outcomes. Of particular relevance is the introduction and use of medical treatment of EP using methotrexate during the study period. As EPs were almost exclusively managed by surgery in the earlier part of the study period, women with surgically managed EP would potentially have contributed to more events as the follow-up time was longer. It is possible that this could have introduced some bias. We have tried to take this into account by including the year of pregnancy event in the final multivariate models. Any study of fertility behaviour is bound to be flawed if it does not take account of contraceptive practice. We had no means of excluding women who were not intending to be pregnant or spacing their next pregnancy and therefore had to make the assumption that contraception was more likely in women with previous live birth and termination than those with spontaneous first pregnancy loss. We could not find any published literature to support this assumption, but as we have analysed routinely collected population-based data the assumptions about fertility intent are likely to be applicable in the broad majority of cases. There was some data on contraception available in the form of IUCD inserted in secondary care and the analysis of this supported our assumption. Of the 58 cases in which IUCD was inserted, 57 had an initial live birth and one had a termination of her first pregnancy. There were no women with an initial miscarriage or EP who had IUCD inserted. The logic behind assuming that women with a first EP will have similar contraceptive practices as those with an initial miscarriage is that neither group has any living children but both have suffered a spontaneous loss of pregnancy. This characteristic also makes them different from women with a previous live birth or a voluntary termination of pregnancy.

Lastly, analysis of such a large population-based dataset is likely to show statistically significant differences that may or may not be clinically relevant. However, our research was driven by a clear hypothesis based on biological plausibility and our results are consistent with those reported in the literature.

### Comparison With Literature

Previous researchers have reported conflicting results with regard to fertility after an ectopic pregnancy. In France, several prospective studies have been conducted in which women with EP have been identified from regional condition-specific registers [8],[12],[15],[16],[18],[23]. Studies based on the Auvergne registry [11] in France reported that determinants of fertility after ectopic pregnancy included the type of contraception used and the method of treatment for ectopic pregnancy. As this study did not incorporate a comparison group with an intrauterine pregnancy, it is difficult to compare the findings with the current analysis. A second report [22] compared data from two regional registers in France and found that fertility rates following an initial ectopic were higher in Auvergne compared to Lille. A third register-based study from Lille, France [24] concluded that over half of women with an ectopic pregnancy conceived naturally and that key determinants of fertility were more likely to be patient characteristics like age rather than factors related to the ectopic pregnancy itself. This conclusion was consistent with our findings. Hassan and Killick [7] using self-reported data from a survey, reported a 3-fold increase in conception rates following ectopic pregnancy. Thorburn et al. [25] reported that the conception rate following EP in women desiring another pregnancy was 75.9%—a proportion significantly higher than that found in the current analysis. The explanation for this variation may lie in the fact that Thorburn et al. [25] as well as Hassan and Killick [7] only included women desiring another pregnancy in their sample population, whereas we had no means of excluding those who wished to avoid another pregnancy from our analysis. On the other hand, as we only included women who had an ectopic first pregnancy and therefore no live children, the majority could be expected to try to conceive again at some point in time. However, as EP is known to occur more frequently in women who use certain types of IUCDs [26], it is possible that at least some of the women in our cohort were voluntarily avoiding another pregnancy. The rates of intrauterine pregnancy, ectopic pregnancy, and live birth following two ectopic pregnancies as reported by Glock et al. [27] are closer to our findings. The high recurrence rate of ectopic pregnancy has been reported elsewhere. An extensive search of the literature failed to identify any reports of outcomes of a continuing second pregnancy following an initial EP. Live birth rates of over 80% have been reported [12],[13], but few studies had the power to assess other maternal and fetal outcomes.

### The Meaning Of The Study

This report presents a comprehensive overview of reproductive sequelae of EP using routinely collected population-based data. Although compromised fertility following ectopic pregnancy has been suspected previously, this is the first report to present empirical evidence to quantify the reduced chance of fecundity after an EP compared to women who have had an early intrauterine pregnancy loss. This reduction in fertility may be partly explained by the emotional turmoil following EP [28] and reluctance to try for a pregnancy for fear of a repeat EP, but this is unlikely to explain such a large difference. An initial EP does not appear to reduce the likelihood of pregnancy compared to women who have had a live birth—but this may well be due to voluntary birth spacing in a western population in the first few years after giving birth. The variation over the three time periods of the relationship between conceptions following EP and live birth bears further evidence of this voluntary fertility control. While the women with EP, and therefore with no living children were more likely to try and conceive another pregnancy within 2 y in comparison to women already with a baby, this difference was not observed in the 2–6-y period following the first pregnancy event. This indicates that women with a previous live birth were possibly spacing their next pregnancy event at least 2 y after their first delivery. It is interesting to note that the likelihood of a second conception is increased once more in the women with previous EP after 6 y, possibly suggesting a role of assisted conception in those with persistent tubal infertility alongside a sense of achievement of desired family size in women with an initial live birth who now have two live children.

It is, however, reassuring to note that those who do have an ongoing pregnancy following an EP are at no significant higher risk of developing complications than a primigravid woman.

### Clinical Implications

There have been few data on reproductive outcomes following ectopic pregnancy compared to successful pregnancy outcomes or other types of pregnancy loss. The results of this study will help clinicians to counsel women with EP—both at the time of initial diagnosis and treatment, as well as later when they attend for antenatal care. The reduced chance of a pregnancy and increased risk of a second ectopic following EP indicates the need for a fertility follow-up in women who are keen to conceive. This would include pre-conception care, advice to seek expert consultation should they wish to start a family, and consideration of an early pregnancy scan to confirm an intrauterine gestation. Overall, the results from this study are broadly reassuring in establishing that obstetric outcomes following ectopic pregnancy are no worse than those in women in their first pregnancy. As such, it is unnecessary to monitor these women more closely during an ongoing intrauterine pregnancy than is customary in primigravid women.

### Future Research

Future large population-based studies on reproductive outcomes after ectopic pregnancy need to explore the influence of the site of the ectopic pregnancy (tubal versus non-tubal) and to incorporate the effect of key confounders like smoking. Given the increasing use of medical treatment for ectopic pregnancy, there is a need to determine the effect of medical versus surgical management and conservative versus radical surgical management of EP on future reproduction. Trials are underway to assess these effects [29].

### Conclusions

Compared to women who have a live birth, women with an initial EP are 2.76 times more likely to conceive a second time within 2 y and just as likely between 2 and 6 y. An ectopic first pregnancy reduces the probability of a second conception leading to clinical pregnancy within the next 2 y in comparison with an initial miscarriage. In those who do conceive, over three-quarters of women go on to have a livebirth. They also run much higher risks of repeat EP. In the next ongoing pregnancy after an ectopic, women run significantly higher risks of preeclampsia, preterm delivery, and emergency caesarean delivery compared to those with a previous live birth. However, these risks are not significantly higher compared to primigravidae or those who have experienced other types of early first pregnancy loss.

## Supporting Information

Text S1 STROBE checklist for reporting observational studies. (DOC)

## Abbreviations

AHR: adjusted hazard ratio
AOR: adjusted odds ratio
EP: ectopic pregnancy
IUCD: intrauterine contraceptive device
OR: odds ratio
