# Supplementary material for: Reproductive Outcomes Following Ectopic Pregnancy: Register-Based Retrospective Cohort Study
Source: PLoS Med. 2012 Jun 19;9(6):e1001243. doi: 10.1371/journal.pmed.1001243 (PMC3378618; doi:10.1371/journal.pmed.1001243)
Supplement: Text S1 — (DOC) [file pmed.1001243.s001.doc]

**Reproductive outcomes following ectopic pregnancy: register based retrospective cohort study**

Supplemental file S1: STROBE Statement

Checklist of items that should be included in reports of cohort studies

|  | Item No | | Recommendation | Location within manuscript |
| --- | --- | --- | --- | --- |
| **Title and abstract** | 1 | | (*a*) Indicate the study’s design with a commonly used term in the title or the abstract | Title & Abstract: Line 51 |
|  |  |  | (*b*) Provide in the abstract an informative and balanced summary of what was done and what was found | Abstract: Lines 52 - 71 |
| Introduction | | | |  |
| Background/  rationale | 2 | | Explain the scientific background and rationale for the investigation being reported | Introduction: Lines 83 - 101 |
| Objectives | 3 | | State specific objectives, including any prespecified hypotheses | Introduction: Lines 101-103 |
| Methods | | | |  |
| Study design | 4 | | Present key elements of study design early in the paper | Methodology: Line 106 |
| Setting | 5 | | Describe the setting, locations, and relevant dates, including periods of recruitment, exposure, follow-up, and data collection | Methodology Lines  106 -115 and 127 - 133 |
| Participants | 6 | | (*a*) Give the eligibility criteria, and the sources and methods of selection of participants. Describe methods of follow-up | Methodology: Lines 109 - 115 |
|  |  |  | (*b*) For matched studies, give matching criteria and number of exposed and unexposed | Not applicable |
| Variables | 7 | | Clearly define all outcomes, exposures, predictors, potential confounders, and effect modifiers. Give diagnostic criteria, if applicable | Methodology: Lines 118 - 125 |
| Data sources/ measurement | 8* | | For each variable of interest, give sources of data and details of methods of assessment (measurement). Describe comparability of assessment methods if there is more than one group | Methodology: Lines 135 - 151 . |
| Bias | 9 | | Describe any efforts to address potential sources of bias | The only possible source of bias could be misclassification of variables as routinely collected data are used. We think that the large dataset should compensate for that. |
| Study size | 10 | | Explain how the study size was arrived at | All available data were included.  Power calculation: lines 182 -194. |
| Quantitative variables | 11 | | Explain how quantitative variables were handled in the analyses. If applicable, describe which groupings were chosen and why | Methodology: Statistical analysis: Lines 160 - 173 |
| Statistical methods | 12 | | (*a*) Describe all statistical methods, including those used to control for confounding | Statistical analysis: Lines 160 - 173 |
|  |  |  | (*b*) Describe any methods used to examine subgroups and interactions | Methodology: Lines 135 - 147 |
|  |  |  | (*c*) Explain how missing data were addressed | Methodology: Lines 152 - 159 |
|  |  |  | (*d*) If applicable, explain how loss to follow-up was addressed | Not applicable. |
|  |  |  | (*e*) Describe any sensitivity analyses | Methodology Lines 155 - 156 |
| Results | | | |  |
| Participants | 13* | | (a) Report numbers of individuals at each stage of study—eg numbers potentially eligible, examined for eligibility, confirmed eligible, included in the study, completing follow-up, and analysed | Results: Lines 176 - 177 |
|  |  |  | (b) Give reasons for non-participation at each stage | Not applicable |
|  |  |  | (c) Consider use of a flow diagram | The whole population was selected |
| Descriptive data | 14* | | (a) Give characteristics of study participants (eg demographic, clinical, social) and information on exposures and potential confounders | Table 1 and Results: Lines 177 - 182 |
|  |  |  | (b) Indicate number of participants with missing data for each variable of interest | Table 1 and lines 152 - 159 |
|  |  |  | (c) Summarise follow-up time (eg, average and total amount) | Fig. 1 and results lines 182 - 186 |
| Outcome data | 15* | | Report numbers of outcome events or summary measures over time | Tables 1 & 3 |
| Main results | 16 | | (*a*) Give unadjusted estimates and, if applicable, confounder-adjusted estimates and their precision (eg, 95% confidence interval). Make clear which confounders were adjusted for and why they were included | Table 2, Table 4 |
|  |  |  | (*b*) Report category boundaries when continuous variables were categorized | Methodology |
| Other analyses | 17 | | Report other analyses done—eg analyses of subgroups and interactions, and sensitivity analyses | Results: Lines |
| Discussion | | | |  |
| Key results | 18 | Summarise key results with reference to study objectives | | Discussion: Lines |
| Limitations | 19 | Discuss limitations of the study, taking into account sources of potential bias or imprecision. Discuss both direction and magnitude of any potential bias | | Discussion: Lines |
| Interpretation | 20 | Give a cautious overall interpretation of results considering objectives, limitations, multiplicity of analyses, results from similar studies, and other relevant evidence | | Discussion: Lines |
| Generalis-ability | 21 | Discuss the generalisability (external validity) of the study results | | Discussion: Lines |

| Other information | | |  |
| --- | --- | --- | --- |
| Funding | 22 | Give the source of funding and the role of the funders for the present study and, if applicable, for the original study on which the present article is based | Lines 34 - 35 |
